# Changes in the liver proteome in apoE knockout mice exposed to inhalation of silica nanoparticles indicate mitochondrial damage and impairment of ER stress responses associated with microvesicular steatosis

**DOI:** 10.1007/s11356-022-22179-6

**Published:** 2022-07-29

**Authors:** Kamila Stachyra, Anna Kiepura, Maciej Suski, Magdalena Ulatowska-Białas, Katarzyna Kuś, Anna Wiśniewska, Klaudia Czepiel, Grzegorz Majka, Rafał Olszanecki

**Affiliations:** 1 Chair of Pharmacology, Faculty of Medicine, Jagiellonian University Medical College, 16 Grzegorzecka Street, 31-531 Krakow, Poland; 2 Department of Pathomorphology, Jagiellonian University Medical College, 16 Grzegorzecka Street, 31-531 Krakow, Poland; 3 Chair of Immunology, Faculty of Medicine, Jagiellonian University Medical College, 18 Czysta Street, 31-121 Krakow, Poland

**Keywords:** Silica nanoparticles, Liver microvesicular steatosis, ER stress, Unfolded protein response, Proteomics, ApoE-knockout mice

## Abstract

The adverse effects of air pollution on the cardiovascular system have been well documented. Nonalcoholic fatty liver disease (NAFLD) is an independent risk factor for cardiovascular events. However, the influence of exposure to airborne particles on the development of NAFLD is less recognised. The aim of this study was to investigate the impact of silica nanoparticles (SiNPs) on the development of liver steatosis. We used molecular and proteomic SWATH-MS methods to investigate the changes in the liver proteome of apolipoprotein E-knockout mice (*apoE*^*−/−*^ mice) exposed to SiNPs for 4 months in a whole-body exposure chamber. Exposure to SiNPs evoked microvesicular liver steatosis in *apoE*^*−/−*^ mice. Quantitative liver proteomics showed significant downregulation of ribosomal proteins and endoplasmic reticulum proteins. Gene expression analysis revealed a reduced level of proteins related to endoplasmic reticulum stress. Treatment with SiNPs decreased mitochondrial membrane potential and increased the production of reactive oxygen species in cultured HepG2 cells. This is the first report that inhalation exposure to SiNPs induces microvesicular steatosis and significant changes in the liver proteome in vivo. Our results highlight the important role of silica and point to the ER stress response and mitochondrial dysfunction as potential mechanisms responsible for the increase in fatty liver by SiNPs.

**Supplementary Information:**

The online version contains supplementary material available at 10.1007/s11356-022-22179-6.

## Introduction

Prolonged exposure to inhaled air pollutants in the form of suspended particulate matter (PM), especially their fraction of diameter less than 2.5 µm (PM_2.5_), is an important risk factor for the development of coronary heart disease (Brook et al. 2010; Simkhovich et al. 2009). The core mechanisms of the pro-atherosclerotic action of PMs have been proposed, based on the assumption that PM_2.5_ can pass from the alveoli into the circulation and act directly on the vascular walls, augmenting local inflammation and oxidative stress (Arias-Pérez et al. 2020; Bai and Sun 2016).

Nonalcoholic fatty liver disease (NAFLD) is currently recognized as an independent risk factor for severe cardiovascular events and an important mechanistic stimulus for the acceleration of atherosclerosis (Hyogo et al. 2014; Stahl et al. 2019). Several epidemiological studies have pointed to an association between the severity of PM exposure and the risk of metabolic syndrome (Clementi et al. 2019). Moreover, in animal experiments, exposure to PM has recently been shown to exacerbate fatty liver, and this may represent an important causal link mechanism between cardiovascular effects and exposure to air pollutants (Ding et al. 2019; Reyes-Caballero et al. 2019). However, despite the general indication of an augmentation of pro-inflammatory and pro-oxidative processes, the molecular mechanisms responsible for the exacerbation of fatty liver caused by inhaled PM are not clear.

Silica nanoparticles (SiNPs) constitute an important part of the inorganic component of PM, especially in cities with high industrial pollution (Mol et al. 2020; Zhang et al. 2017). Most of the data on the harmful effects of silica on the human body come from research on its crystalline varieties, but as numerous observations show, the amorphous form of silica can also be found in airborne particles (ATSDR 2019). In our research, we dealt with the amorphous form, which is still largely underrepresented in the literature pertaining to the biological effects of air pollution.

Recently, in the apolipoprotein E knockout mouse model (*apoE*^*−/−*^ mouse), we have shown that SiNPs are distinguished from other inhaled pollutants by their potent pro-atherosclerotic action, which was partly based on the skewing of polarisation monocytes towards the M1 phenotype and augmentation of inflammatory reactions in the plaque (Stachyra et al. 2021).

In this study, we investigate the impact of inhaled exposure to SiNPs on the development of liver steatosis in *apoE*^*−/−*^ mice, and we try to identify the potential molecular pathways of action of SiNPs in the liver by applying the SWATH-MS (*sequential window acquisition of theoretical fragment ion spectra mass spectrometry*) proteomic quantitative method to a comprehensive analysis of changes in the liver proteome of *apoE*^*−/−*^ mice fed a diet with moderate fat content.

## Materials and methods

### Silica nanoparticles preparation for in vivo experiment

Amorphous silicon oxide (SiO_2_) was obtained from US Research Nanomaterials, Inc (Houston, TX, USA) in the form of an aqueous suspension (25 wt%). The particle size of silica varies from 5 to 35 nm. The stock suspension of silica nanoparticles (SiNPs) was diluted in distilled water to obtain a final concentration of 0.6 mg/mL for the in vivo experiment. The selected properties of the aqueous suspension of SiNPs were published (Stachyra et al. 2021).

### Animal experiments

All animal procedures were in accordance with the guidelines of Directive 2010/63/EU of the European Parliament on the protection of animals used for scientific purposes and were approved by the Jagiellonian University Ethical Committee on Animal Experiments (approval number 105/2018).

Twenty-four female apolipoprotein E-knockout mice on the C57BL/6 J background were obtained from Charles River (Calco, Italy). The mice were maintained on reversed 12-h dark/12-h light cycles in air-conditioned room (22.5 ± 0.5 °C, 50 ± 5% humidity) with free access to feed and water. At 8 weeks of age, the animals were put on a 10% fat-containing diet (Ssniff, Germany) and randomly divided into two groups: control (*n* = 12) and SiNP-treated group (*n* = 12). Mice were exposed to aqueous suspension of silica nanoparticles by nebulisation using the Aeroneb Pro Nebulizer (Aerogen, Galway, Ireland). Inhalation was performed 5 h/day, 5 days/week for 16 weeks in a whole-body exposition chamber that allowed free movement, as described before (Stachyra et al. 2021). The aerosol dose concentration achieved in the chamber was approximately 400 µg/m^3^. The control group was exposed to distilled water under the same conditions. After the end of the experimental period, mice were injected with 1000 IU of fraxiparine (Sanofi-Synthelabo, Paris, France) and sacrificed in a carbon dioxide chamber. Blood and liver samples were collected for further investigation.

### Histology of the liver

Liver tissue samples were fixed in formalin, routinely processed, and embedded in paraffin. Sections were prepared from paraffin blocks and stained with the hematoxylin–eosin (HE) method. Then, the slides were microscopically observed by a pathologist blinded to all experimental groups. The presence of steatosis and the type of steatosis, macrovesicular or microvesicular, in the samples were evaluated. Three independent sections in three different fields of view were assessed for each animal. The overall percentage of hepatocytes containing fat in the cytoplasm (as small droplets or one large drop) was estimated according to SAF (Steatosis Activity Fibrosis) scoring system for NAFLD: S0: < 5% of hepatocytes containing fat; S1: 5–33% of hepatocytes containing fat; S2: 34–66% of hepatocytes containing fat; and S3: > 66% of fat-containing hepatocytes.

### Biochemical methods

Blood was centrifuged at 1000 × g and 4 °C for 10 min. Plasma levels of alanine aminotransferase (ALT) and aspartate aminotransferase (AST) were evaluated using commercially available kits Reflotron GPT and Reflotron GOT (Roche, Basel, Switzerland) by the Reflovet Plus analyser (Roche, Basel, Switzerland). The content of triglycerides in the liver was measured using the Triglyceride Colorimetric Assay Kit (Cayman Chemical, Ann Arbor, MI, USA), according to the manufacturer’s instructions.

### Real-time PCR

Total RNA was isolated from the liver using the ReliaPrep RNA Tissue Miniprep System (Promega, Madison, WI), according to the manufacturer’s instructions. Reverse transcription was carried out using a High-Capacity Reverse Transcription Kit (Applied Biosystems, Foster City, CA). Quantitative real-time PCR was performed using the Bio-Rad CFX96 Touch™ Real-Time PCR System with GoTaq® qPCR Master Mix (Promega, Madison, WI). The primers for *Atf4* (NC 000,081.6), *Ddit3* (gen for CHOP; NC 000,076.6), *Hspa5* (gen for GRP78; NC 000,068.7), *Hsp90b1* (gen for GRP94; NC 000,076.6), *Mfn2* (NC 000,070.6), and *Sod2* (NC 000,083.6) were obtained from Bio-Rad (Hercules, CA, USA). Data were analysed by the Bio-Rad CFX Maestro Software using 2^−ΔΔCt^ method. The expression of *Gapdh* (NC 000,072.6) was used to normalize the data.

### Cell culture experiments

Human liver cancer cells (HepG2) were obtained from ATCC (HB-8065) and cultured in DMEM medium supplemented with penicillin-steptomycin (Gibco) and 10% foetal bovine serum (Gibco). Cells were grown at 37 °C in a humidified atmosphere containing 5% CO_2_ and 95% air to achieve the appropriate confluency. Based on the results of the cytotoxicity assays, cells were treated with silica nanoparticles at a concentration of 100 µg/mL for 24 h. The working solutions of SiNPs were prepared freshly prior to each in vitro experiment by dilution stock suspension in PBS. Cells between 3 and 5 passages were used for experiments.

### MTT assay

The viability of HepG2 cells was evaluated using the MTT assay. Briefly, cells were seeded at a density of 2 × 10^4^/well in 96-well plates. After 24 h of incubation with SiNPs, MTT (10 mg/mL in PBS) was added to the cells and incubated at 37 °C for 1 h. Next, the formed formazan crystals were dissolved in dimethyl sulfoxide, and the absorbance was measured at 540 nm using the Synergy H1 microplate reader (BioTek Instruments, Inc., Winooski, VT).

### LDH assay

The measurement of lactate dehydrogenase (LDH) released from HepG2 cells was performed to assess the cytotoxicity of SiNPs. The experiment was carried out using the Pierce LDH Cytotoxicity Assay Kit (Thermo Scientific, Waltham, MA), according to the manufacturer’s instructions. Absorbance was measured at 490 nm and 680 nm using the Synergy H1 microplate reader (BioTek Instruments, Inc., Winooski, VT).

### Mitochondrial membrane potential

The mitochondrial membrane potential was determined using the MitoProbe™ JC-1 Assay Kit (ThermoFischer), according to the manufacturer’s instructions. Cells were incubated with 10 µM JC-1 solution for 30 min at 37 °C. The fluorescence signal was measured by flow cytometry (FACSCantoII, Becton Dickinson, Franklin Lakes, NJ).

### Mitochondrial superoxide measurement

The production of superoxide anion in HepG2 cells was detected using the MitoSOX Red Mitochondrial Superoxide Indicator (Molecular Probes, Eugene, OR, USA), according to the manufacturer’s instructions. Fluorescence intensity measurement was performed by Synergy H1 microplate reader (BioTek Instruments, Inc., Winooski, VT).

### Sample preparation for LC–MS/MS analysis

The liver subcellular fractions were processed with the same sample preparation protocol. The isolated mitochondria were lysed in buffer to tissue ratio of 10 (w/v) with 2% SDS, 50 mM DTT in 0.1 M Tris–HCl pH 7.6, vortexed, incubated at 95 °C for 5 min, and clarified by centrifugation at 14,000 g for 10 min. Cytosolic fractions were concentrated and purified by overnight acetone precipitation (1:6 sample to acetone ratio), then centrifuged at 14,000 g for 10 min. The obtained protein pellets were then lysed in lysis buffer similarly to mitochondrial specimens. Before protein digestion, the total protein concentration in the collected lysates was determined by the WF assay (Wiśniewski and Gaugaz 2015). Next, a volume containing 70 µg of total protein was transferred to Microcon-30-kDa centrifugal filter units (Merck, Darmstadt, Germany), denaturated with 8 M urea in 0.1 M Tris–HCl pH 8.5, and digested to peptides using the filter-aided sample preparation (FASP) protocol (Wiśniewski et al. 2009). Proteins were alkylated with iodoacetamide and cleaved with trypsin (Thermo Scientific, Waltham, MA) with the enzyme to protein ratio of 1:50. The digestions were carried out overnight at 50 mM Tris–HCl pH 8.5 at 37 °C. After digestion, peptide yields were determined by the WF assay, and aliquots containing an equal amount of total peptides were desalted in C_18_ Ultra-Micro SpinColumns (Harvard Apparatus, Holliston, MA). The samples were then concentrated in a volume of ~ 5 µL. For project-specific spectral library preparation (separately for mitochondrial and cytosolic fractions), an equal amount of peptides from all samples included in the analysis were combined and subjected to the fractionation protocol. HpH fractionation on C_18_ Micro SpinColumns (Harvard Apparatus, Holliston, MA) was performed in 50 mM ammonium formate buffer (pH 10) with 12 consecutive injections of eluent buffer, comprising 5, 10, 12.5, 15, 17.5, 20, 22.5, 25, 27.5, 30, 35, and 50% acetonitrile in 50 mM ammonium formate buffer (pH 10), collected by centrifugation (300 × g, 2 min) and dried in a speedvac concentrator (Eppendorf, Hamburg, Germany). In this way, the peptides were distributed across 12 HpH fractions separately for both subcellular fractions and analysed by LC–MS/MS in DDA acquisition mode for library generation. Prior the analysis, all samples and library peptide fractions were solubilized in 0.1% formic acid in 5% acetonitrile at a concentration of 0.5 µg/µl and spiked with the iRT peptide mix (Biognosys, Schlieren, Switzerland) for normalisation of retention time.

### Liquid chromatography-tandem mass spectrometry

Peptides (1 µg) were injected into a PepMap100 RP C18 75 µm i.d. × 25 cm column (Thermo Scientific, Waltham, MA) via a trap column PepMap100 RP C18 75 µm i.d. × 2 cm column (Thermo Scientific, Waltham, MA). For library generation, each peptide fraction was separated using a 98 min 1 to 40% B phase linear gradient (A phase—2% ACN and 0.1% FA; B phase—80% ACN and 0.1% FA) operating at a flow rate of 300 nL/min on an UltiMate 3000 HPLC system (Thermo Scientific, Waltham, MA) and applied to a TripleTOF 6600 + (Sciex, Framingham, MA) mass spectrometer. The main working nano-electrospray ion source (Optiflow, Sciex, Framingham, MA) parameters were as follows: ion spray voltage 3 kV, interface heater temperature (IHT) 200 °C, ion source gas 1 (GS1) 10, and curtain gas (CUR) 25. For DDA acquisition, spectra were collected in full scan mode (350–1400 Da), followed by 100 CID MS/MS scans of 100 most intense precursor ions from the preceding survey full scan exceeding 100 cps intensity under dynamic exclusion criteria. Samples analysed in SWATH acquisition mode were separated using a 63 min 1 to 40% B phase linear gradient at a flow rate of 300 nL/min with the same ion source settings. For SWATH acquisition, spectra were collected in full scan mode (400–1250 Da), followed by 100 SWATH MS/MS scans using a variable precursor isolation window approach, with m/z windows ranging from 6 to 90 Da.

### Mass spectrometric raw data analysis, spectral library generation, and SWATH quantitation

DDA data were searched against the mouse UniProt database (release 2021_01_04, 17,056 entries) and the MaxQuant Contaminants list (245 entries) using the Pulsar search engine implemented in the Spectronaut software (Biognosys, Schlieren, Switzerland) (Bruderer et al. 2015) with default parameters (± 40 ppm mass tolerance at the MS1 and MS2 level, mutated decoy generation method, trypsin enzyme specificity). Deep learning assisted iRT regression was set as the iRT reference strategy for RT to iRT calibration with minimum *R*^2^ set to 0.8. The peptide, protein, and PSM FDR were set to 1%. The library was generated using 3–6 fragment ions per precursor.

The project-specific library was be then used to analyse the SWATH data in Spectronaut (Biognosys, Schlieren, Switzerland). Data were filtered by 1% FDR at the peptide and protein level, while quantitation and interference correction were performed at the MS2 level. Protein grouping was performed based on the ID picker algorithm (Zhang et al. 2007). Single hit proteins were excluded from the final results. Protein quantities were calculated by averaging the respective peptide intensities, while the latter were obtained as mean precursor quantities. The protein coefficients of variation (CVs) were calculated based on the summed intensities of their respective peptides. Data were normalized by global regression strategy, while statistical testing for differential protein abundance was done using *t* tests with multiple testing correction after Storey (Storey 2002). The LC–MS data, library, results’ report, and the Spectronaut project have been deposited to the ProteomeXchange Consortium via the PRIDE partner repository (Vizcaíno et al. 2014) with the dataset identifier PXD028547.

Functional grouping and pathway annotations were performed using ClueGO (Bindea et al. 2009) in the Cytoscape 3.7.2 software environment (Shannon et al. 2003). CORUM-3.0 (release 03.09.2018), KEGG (release 17.02.2020), REACTOME (release 17.02.2020), and WikiPathways (release 17.02.2020) ontologies/pathways were used in the analysis. The enrichment results were validated by a two-sided enrichment/depletion geometric statistical test with Bonferroni step down as a *p* value correction method. The minimum and maximum GO levels were set as 1 and 4, respectively, with the cluster criteria of minimum 5 genes constituting a minimum of 2% of the GO term. The kappa score threshold was set as 0.4.

### Statistical analysis

Biochemical and PCR data, as well as in vitro results, were analysed using GraphPad Prism 8.3.0 (GraphPad Software Inc., San Diego, CA). The results were presented as mean ± standard error of measurement (SEM). The statistical significance of the differences between the groups was analysed using the unpaired *t* test or the Mann–Whitney test, depending on the results of the normality test. Values of *p* < 0.05 were considered statistically significant.

## Results

### Body weight

All animals completed the study protocol. The mean body weight of the control and silica-treated mice did not differ (24.37 ± 2.66 g vs. 24.50 ± 2.80 g, NS). Also, the food intake did not differ between the groups.

### Impact of silica nanoparticles on liver histology and biochemical markers of liver injury

**Fig. 1:**
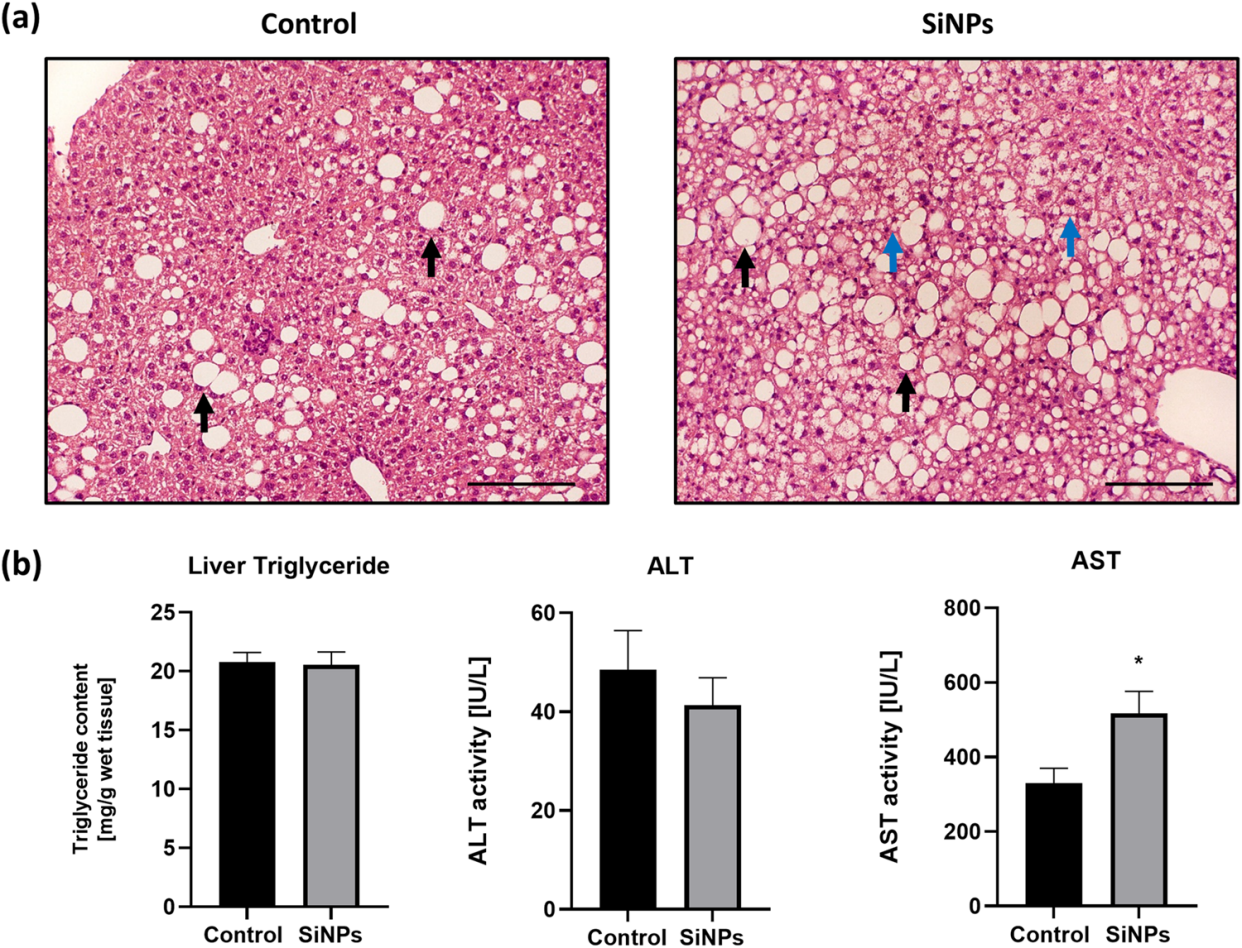
Influence of silica nanoparticles on liver tissue histology and biochemical markers in apoE^*−*/*−*^ mice. Representative pictures of HE-stained liver sections showing signs of macrovesicular (black arrows) and microvesicular (blue arrows) steatosis (**a**). The level of ALT and AST in plasma, as well as the content of TG in the liver (**b**). Data presented as mean ± SEM; *p < 0.05 as compared to the control; *n* = 6–8 per group. Scale bar = 50 µm

Histological examination of liver tissue revealed a mixed type of steatosis in the SiNP-treated group: macrovesicular and microvesicular steatosis was present in 50% up to 70% of hepatocytes. For comparison, the cytoplasm of hepatocytes in control mice had signs of macrovesicular steatosis between 40%, focally up to 60% of hepatocytes. Plasma AST level was significantly elevated in SiNP-treated mice, while plasma ALT and liver triglyceride content did not differ between the groups (Fig. 1). Exposure to silica did not change plasma lipid concentrations, as previously indicated (Stachyra et al. 2021).

### Effect of silica nanoparticles on liver proteomic profile

DDA mass spectrometry measurements resulted in the identification of 49 543 and 32 338 proteotypic peptides, which allowed for the preparation of the spectral library comprising 5 620 and 3 788 protein groups (including 799 and 627 single hit proteins) for mitochondrial and cytosolic fractions, respectively. The libraries were then used to analyse SWATH datasets with Spectronaut. Spectral library recovery was 59.9% and 67.1% with data completeness among protein group profiles of 91.3% and 91.3% for mitochondrial and cytosolic fractions, respectively (Supplemental Fig. 1). Median protein group CVs were calculated in the range of 13.4 to 17.1% for all experimental groups in both fractions (Supplemental Fig. 1), which allowed for the estimation of significant quantitative cut-off for absolute 1.4-fold change (statistical power 95% and 94% for mitochondrial and cytosolic fractions, respectively). The estimates of the reproducibility of LC–MS analyses and quantitation quality are collected in Supplementary Figure 2.

**Fig. 2:**
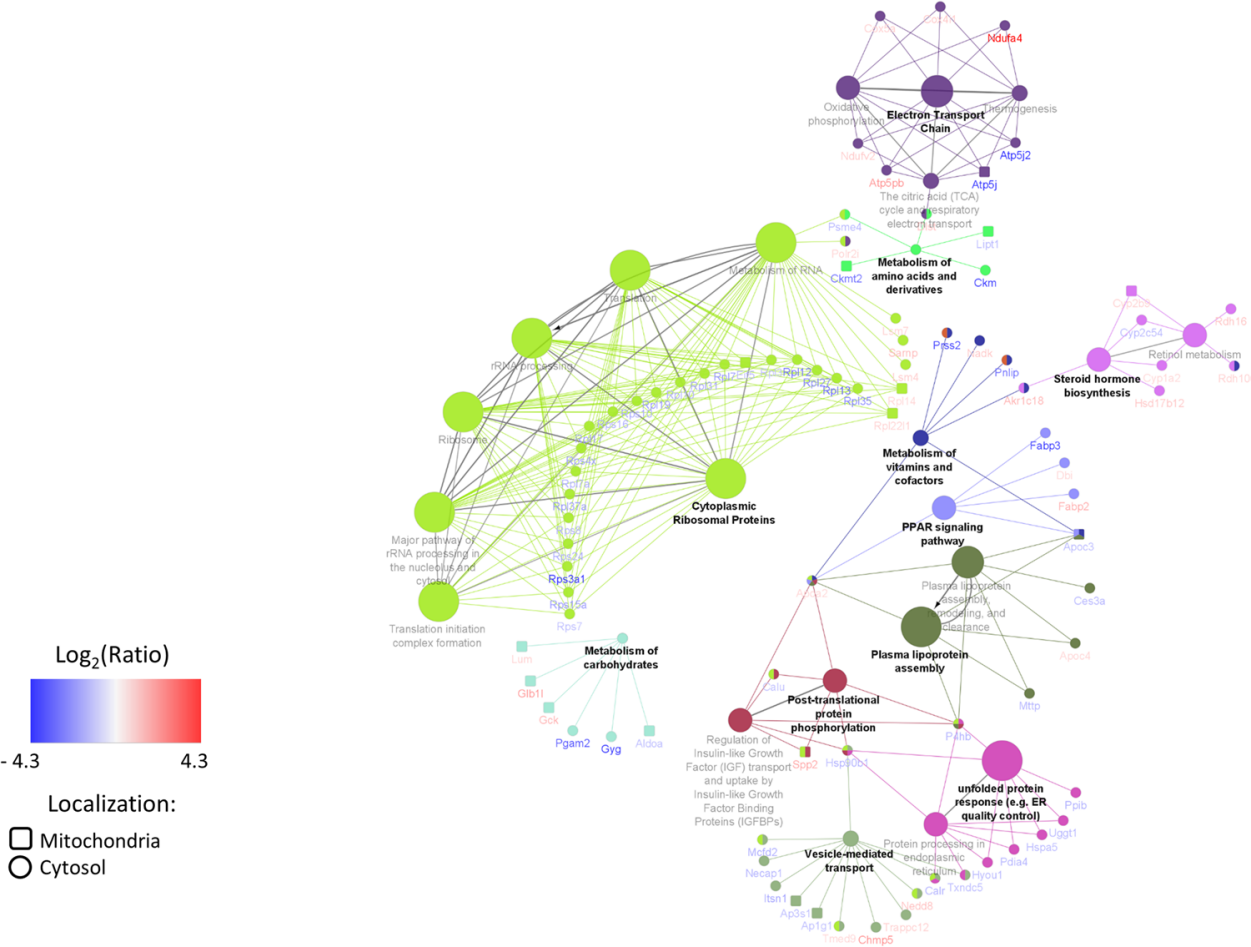
Proteomic analysis of the liver in apoE^*−*/*−*^ mice. The ClueGO network presents proteins from mitochondrial and cytosolic fractions, as well as protein-related pathways in the liver affected by SiNPs

**Fig. 3:**
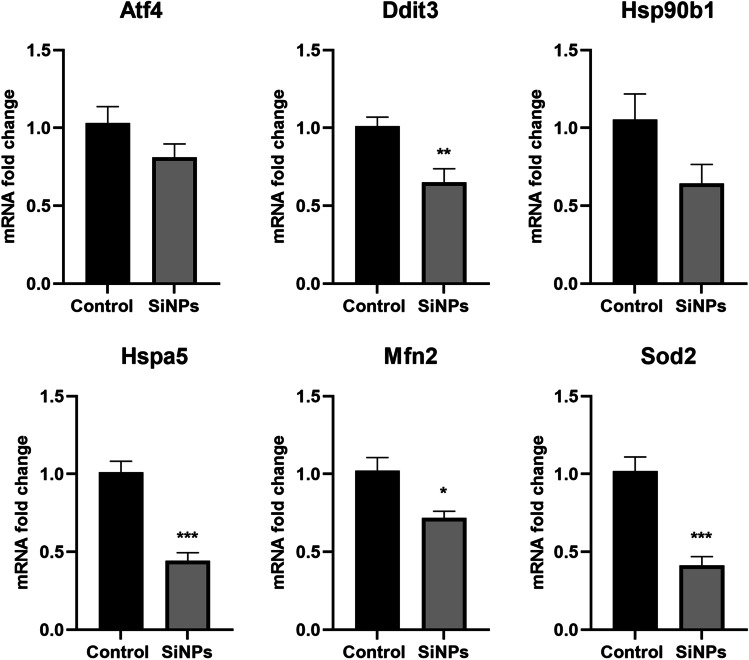
Expression of the genes Atf4, Ddit3 (CHOP), Hspa5 (GRP78), and Hsp90b1 (GRP94) related to ER stress, as well as Mfn2 and Sod2 genes related to mitochondria. Data presented as mean fold change ± SEM; *p < 0.05, **p < 0.01, ***p < 0.001 as compared to the control; *n* = 6 per group

On average, the acquired data allowed for the identification and quantitation of 3082 and 2325 protein groups of which 40 and 130 were significantly regulated in SiNP-treated apoE^*−*/*−*^ mice liver mitochondria and cytosol, respectively (Supplemental Table 1). As expected, many metabolic enzymes were regulated, including those engaged with carbohydrates, aminoacids, vitamins, and lipoprotein turnover (Fig. 2). Interestingly, we identified mitochondrial and endoplasmic reticulum proteins to be affected by SiNPs, with simultaneous profound repression of the protein translation machinery (Fig. 2). PCR analysis confirmed the proteomic results and revealed changes in the expression of genes related to maintaining the mitochondrial network, endoplasmic reticulum stress (ER stress), and unfolded protein response (UPR). Furthermore, we observed a potent decline in *Sod2* expression in the liver. Changes in the expression of selected proteins were correlated with changes in the expression of their mRNA in the liver (Fig. 3).

### Impact of silica nanoparticles on HepG2 cell line

**Fig. 4:**
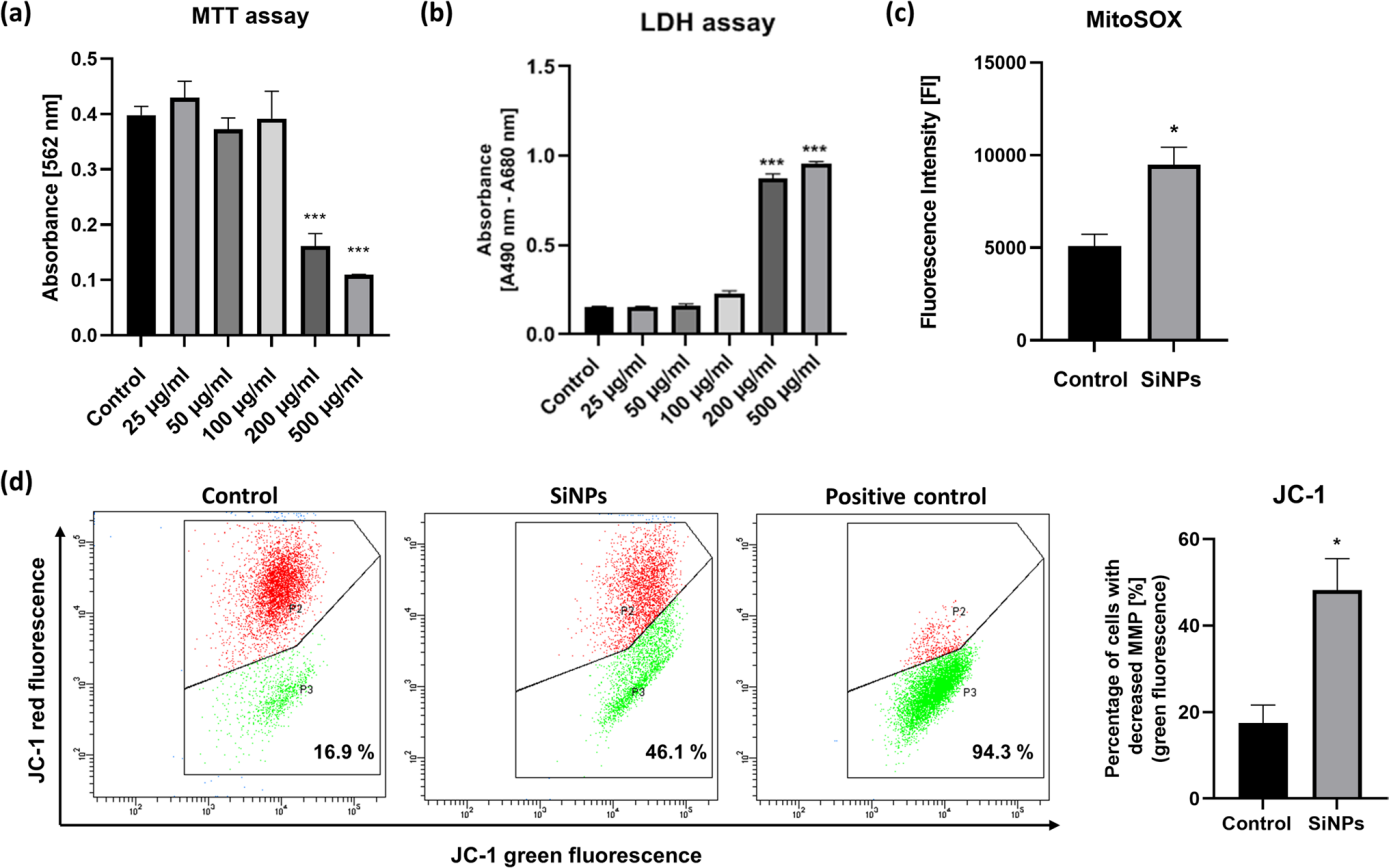
Effects of silica nanoparticles on HepG2 cells. Cell viability after stimulation with various concentrations of SiNPs for 24 h (**a**). LDH release after 24-h exposure to SiNPs (**b**). Mitochondrial superoxide production in cells treated with SiNPs (100 µg/ml) for 24 h (**c**). Flow cytometry analysis of the decrease in mitochondrial potential in cells treated with SiNPs (100 µg/ml) for 24 h; CCCP (carbonyl cyanide 3-chlorophenylhydrazone) was used as a positive control (**d**). Data presented as mean ± SEM; *p < 0.05, ***p < 0.001; *n* = 3–4 per group

We found that SiNP concentrations up to 100 µg/mL did not significantly affect HepG2 cell viability, as evidenced by MTT and LDH assays (Fig. 4). Simultaneously, SiNPs at a dose of 100 µg/mL caused a decrease in mitochondrial membrane potential and increased superoxide anion production by mitochondria (Fig. 4).

## Discussion

The main finding of our research is that inhaled silica nanoparticles evoked liver injury accompanied by microvesicular steatosis in the model of *apoE*^*−/−*^ mice fed with a moderate fat content diet. Although the harmful effects of air particulate matter on the respiratory and cardiovascular systems have been well evidenced, the impact of air pollution on the liver condition is less recognized. The relationship between exposure to air pollution and fatty liver has been shown in several recent epidemiological studies, and a causal relationship has been proved by several studies in animal models (Guo et al. 2022; Xu et al. 2019). However, neither the main pollutant component responsible for the aggravation of fatty liver nor the leading mechanism of its action has been identified. Our study identifies silicon oxide nanoparticles, an important inorganic component of pollutants, as a factor responsible for microvesicular steatosis-related liver damage.

The *apoE*^*−/−*^ mice represent a recognized model for studying the mechanisms of atherosclerosis (Lo Sasso et al. 2016). In our study, we used female *apoE*^*−/−*^ mice, which have been shown to constitute a more homogenous model for in vivo studies compared to male *apoE*^*−/−*^ mice (Marek et al. 2017). Importantly, with the right choice of diet, this model enables the achievement of various degrees of fatty liver. Therefore, *apoE*^*−/−*^ mice have been used in several studies on the mechanisms of liver damage associated with steatosis (Feng et al. 2019; Nasiri-Ansari et al. 2021).

Quantitative proteomics is a well-established method of holistically analysing the protein composition of a tissue or organ and helps to pre-identify the molecular mechanisms involved in the development of pathology. In our research, we have explored for the first time the impact of inhaled silica nanoparticles on the liver proteomic profile in the *apoE*^*−/−*^ mice model.

In our research, we used the SWATH-MS method, which due to its high sensitivity, reproducibility, and consistency becomes these days the most reliable for proteome change quantification (Gillet et al. 2012). In our setting, the inhalation of SiNPs caused significant changes in the liver proteome profile of the *apoE*^*−/−*^ mice. The most striking appeared to be the changes in proteins involved in mitochondrial function (oxidative phosphorylation, respiratory electron transport), as well as the protein translation machinery and the unfolded protein response.

Recent reports indicate that mitochondrial dysfunction plays an important role in the development of microvesicular liver steatosis (Rabinowich and Shibolet 2015; Tandra et al. 2011). Microvesicular steatosis is characterized by the presence of small intrahepatic vesicles containing fat that do not displace the nucleus. According to the literature, microvesicular steatosis is correlated with the severity of NAFLD and contributes to a worse prognosis than macrovesicular steatosis (Oleszczuk et al. 2007). Importantly, the presence of microvesicular steatosis has been found to be associated with toxic mitochondrial injury caused by some chemical substances or drugs (Rabinowich and Shibolet 2015; Satapathy et al. 2015). In our model, we observed an elevated expression of cytochrome c oxidase subunits (NDUFA4, COX4I1, COX5A) in the liver. These proteins belong to complex IV of the respiratory chain and drive oxidative phosphorylation. Furthermore, we observed upregulation of superoxide dismutase 1 (SOD1) in the liver. According to some studies, SOD1 has been implicated in mitochondrial damage (Guareschi et al. 2012). However, the overexpression of SOD1 in our experimental model is likely a compensatory mechanism. Importantly, in our hands, the detrimental effect of SiNPs on mitochondria was also observed in vitro, in a cell culture model. HepG2 cell line has commonly been used to investigate the potential mechanisms related to mitochondrial damage after exposure to nanoparticles (Lee et al. 2021; Sun et al. 2011). Using this model, we found that silica nanoparticles, at concentration, which did not affect cell viability, strongly impaired mitochondrial function, as evidenced by a decrease in mitochondrial membrane potential and increased superoxide anion production. Moreover, the potent decline in SOD2 expression in the liver of apoE mice can also cause an excessive accumulation of superoxide anion leading to mitochondrial injury. Collectively, our results suggest that mitochondrial damage, revealed by disrupted oxidative phosphorylation and increased production of reactive oxygen species, may represent an important factor involved in the development of microvesicular steatosis by silica nanoparticles. In addition, our findings did not show significant plasma lipid changes in mice, and thus implies that exposure to silica revealed the molecular mechanisms of microvesicular steatosis, associated with impaired mitochondrial and ribosomal function, not directly related to lipid/lipoprotein metabolism.

Interestingly, inhaled SiNPs broadly decreased the expression of all ribosomal proteins, as well as proteins involved in the unfolded protein response in the liver. Endoplasmic reticulum stress and unfolded protein response signalling have been demonstrated to be dysregulated in multiple disorders, including liver steatosis (Henkel and Green 2013; Song and Malhi 2019). The primary regulator of the unfolded protein response is HSPA5, which exhibits chaperone activity and plays a key role in protein folding and quality control in the endoplasmic reticulum. Studies using mouse models reported that loss of HSPA5 exacerbates fatty liver disease, while overexpression of HSPA5 reduces liver steatosis (Henkel and Green 2013). In our study, inhalation of SiNP was associated with downregulation of HSPA5 in the liver; thus, it appears that inhibition of UPR may be an important mechanism in SiNP-induced liver damage. The endoplasmic reticulum and mitochondria are two physically connected organelles, and some mitochondrial proteins can modulate the response to ER stress. Mitofusin2 (Mfn2) is a key protein that participates in mitochondrial fusion, which has been observed to regulate the ER stress and UPR pathways. Furthermore, studies report that Mfn2 deficiency affects mitochondrial function. Our results show that changes in the liver proteome and impaired ER stress are linked to decreased expression of Mfn2. These observations may suggest that mitochondria and endoplasmic reticulum are the common targets of SiNP action. However, this interesting hypothesis requires further corroboration. In addition to liver proteome investigation, the exploration of liver transcriptome could be interesting for subsequent studies.

So far, only several in vitro and in vivo studies have been performed to examine the effects of silica nanoparticles using proteomic methods. The study on HepG2 cells reported SiNP-dependent downregulation of ribosomal proteins and upregulation of proteins related to oxidative phosphorylation (Lee et al. 2021). Interestingly, this study concluded that changes in proteins associated with oxidative phosphorylation are subsequent to the increase in the production of reactive oxygen species induced by SiNPs. Another study conducted on cultured lung epithelial cells confirms the reduction of ribosomal proteins after treatment with SiNPs (Okoturo-Evans et al. 2013). In one in vivo study, the influence of intragastric administration of silicon oxide nanoparticles on the proteomic profile of rat liver microsomes has been investigated (Tananova et al. 2015). The authors reported that the administration of SiNPs inhibits the biosynthesis of the HSPA5 protein; thus, our results are consistent with their findings. On the contrary, another report demonstrated that exposure to SiNPs increased the expression of HSPA5 and other markers of unfolded protein response proteins in spermatocyte cells and the human neuroblastoma cell line (Hou et al. 2021; Ren et al. 2020). Hence, we can speculate that exposure to silica nanoparticles alters the UPR but in a different manner, depending on the experimental model.

Obviously, our model shows some limitations. Despite the overall amount of silica in the environmental pollutants is relatively high, the most widespread form of silica in the air is crystalline silica, whereas in our experiment, we used silica nanoparticles in amorphous form. Some studies indicate that amorphous silica nanoparticles can induce toxicity similar to that of crystalline particles and inhalation could also be an important exposure route for amorphous silica (Murugadoss et al. 2017). Nevertheless, transferring our results to human health effects must be cautious. Nanoparticle administration may also pose some important concerns. It is impossible to determine the accurate exposure dose of aerosol inhaled by individual mice using a whole body exposure system. Moreover, as the main route of nanoparticle delivery in whole body exposure is the respiratory tract, it is still significant, yet impossible, to determine the amount of particles that could get to the digestive system (and then possibly circulation). However, it should be noted that despite these limitations, inhalation in a whole-body exposure system allows free movement of animals during the experiment and is one of the best techniques in environmental studies.

## Conclusions

We conclude that inhalation exposure to silica nanoparticles induced microvesicular steatosis and significantly altered the protein profile in the liver of *apoE*^*−/−*^ mice. The biological pathway especially affected by SiNPs refers to endoplasmic reticulum stress and unfolded protein response signalling, as well as to impaired mitochondrial function. Our results highlight the important role of exposure to silicon compounds by inhalation in the development of microvesicular liver steatosis.

## Supplementary Information

Below is the link to the electronic supplementary material.

Supplementary file1 (XLSX 43 KB)

Supplementary file2 (DOCX 2091 KB)

## Data Availability

The LC–MS data, library, results report and Spectronaut project have been deposited to the ProteomeXchange Consortium via the PRIDE partner repository with the dataset identifier PXD028547.
