# Supplementary material for: Changes in the liver proteome in apoE knockout mice exposed to inhalation of silica nanoparticles indicate mitochondrial damage and impairment of ER stress responses associated with microvesicular steatosis
Source: Environ Sci Pollut Res Int. 2022 Jul 29;30(1):699–709. doi: 10.1007/s11356-022-22179-6 (PMC9813169; doi:10.1007/s11356-022-22179-6)

Environmental Science and Pollution Research

**Changes in the liver proteome in apoE knockout mice exposed to inhalation of silica nanoparticles indicate mitochondrial damage and impairment of ER-stress response associated with microvesicular steatosis**

Kamila Stachyra, Anna Kiepura, Maciej Suski, Magdalena Ulatowska-Białas, Katarzyna Kuś, Anna Wiśniewska, Klaudia Czepiel, Grzegorz Majka, Rafał Olszanecki^*^

*Corresponding author: Chair of Pharmacology, Jagiellonian University Medical College, 16 Grzegorzecka Street, 31-531 Krakow, Poland; [rafal.olszanecki@uj.edu.pl](mailto:rafal.olszanecki@uj.edu.pl)

**Supplementary Materials**

**Supplemental Table 1:** Identification of proteins significantly regulated in the liver of SiNPs-treated apoE^-/-^ mice. Cellular localization (local): M – mitochondrial, C – cytosolic.

Supplemental table was provided as spreadsheet file in Microsoft Excel.

**Supplemental Table 2:** Composition of experimental diet administered to mice. Experimental diet for mice (Ssniff, Germany) - diet with 10% fat and long-chain saturated fatty acids.

| **Crude Nutrients [%]** | **Fatty acids [%]** |
| --- | --- |
| Dry matter 95.7 | C 12:0 0.01 |
| Crude protein (N x 6.25) 20.8 | C 14:0 0.34 |
| Crude fat 10.1 | C 16:0 2.52 |
| Crude ash 5.6 | C 16:1 0.25 |
| N free extracts 54.2 | C 17:0 0.12 |
| Starch 35.7 | C 18:0 1.77 |
| Sugar 16.6 | C 18:1 3.68 |
|  | C 18:2 0.25 |
|  | C 18:3 0.05 |
|  | C 20:0 0.01 |
|  | C 20:4 0.02 |

**Energy [MJ/kg]**

Gross Energy (GE) 19.5 [MJ/kg]

Metabolizable Energy (ME) 16.5 [MJ/kg]: 51% from carbohydrates, 21% from fat, 28% from protein

Cholesterol [mg/kg] 95

**Supplemental Figure 1:** Protein group identification summary. Protein group identification details across all of the LC-MS runs (A). Spectral library recovery (B) and data completeness (C). Coefficient of variations for protein groups in experimental conditions (D).


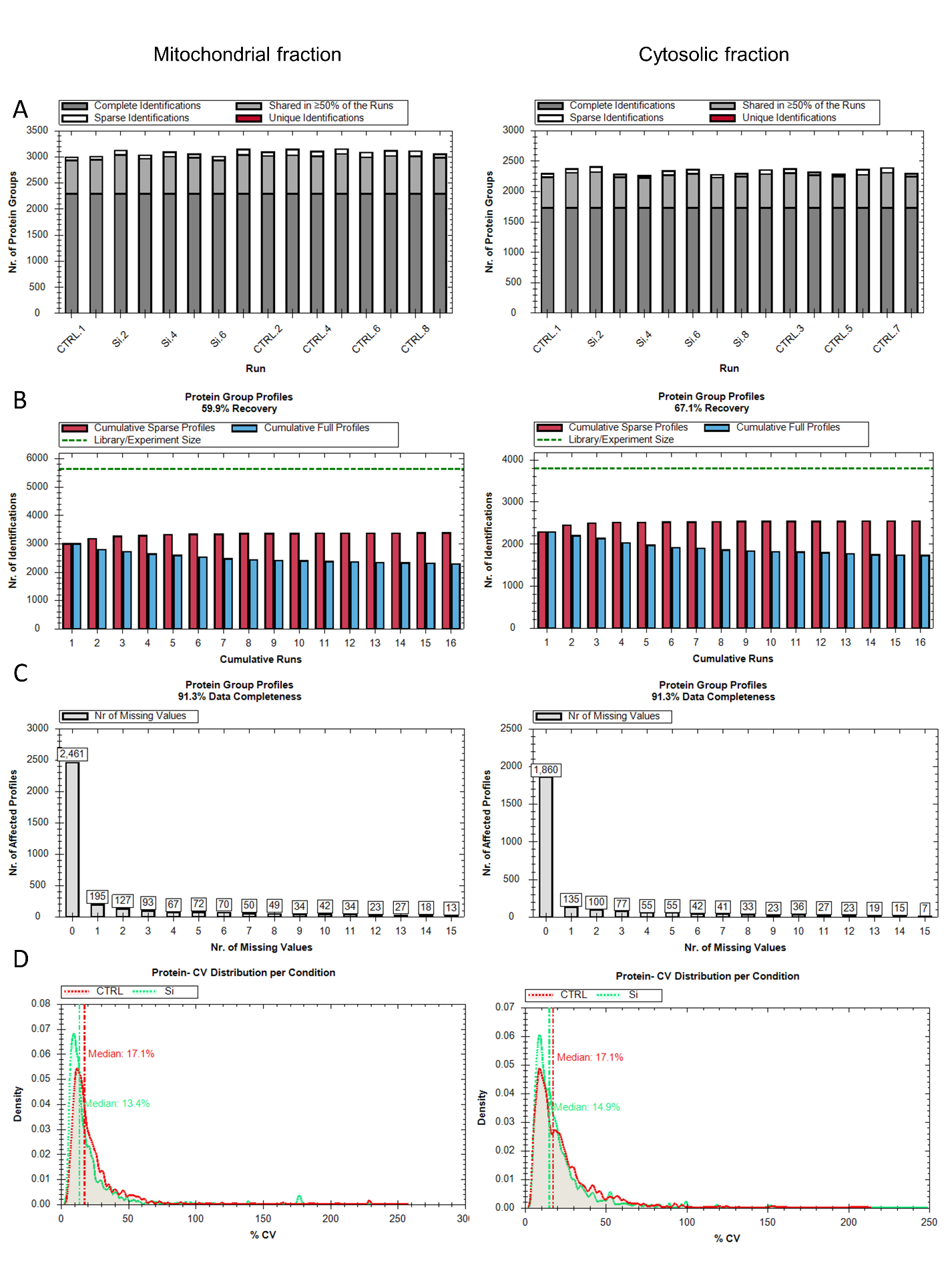


**Supplemental Figure 2:** Protein group quantitation summary. TIC overlay off all LC-MS runs and iRTs elution profiles showed excellent separation reproducibility (A), while normalization of the data allowed for reliable and accurate quantitation, as evidenced by symmetrical histogram (B).


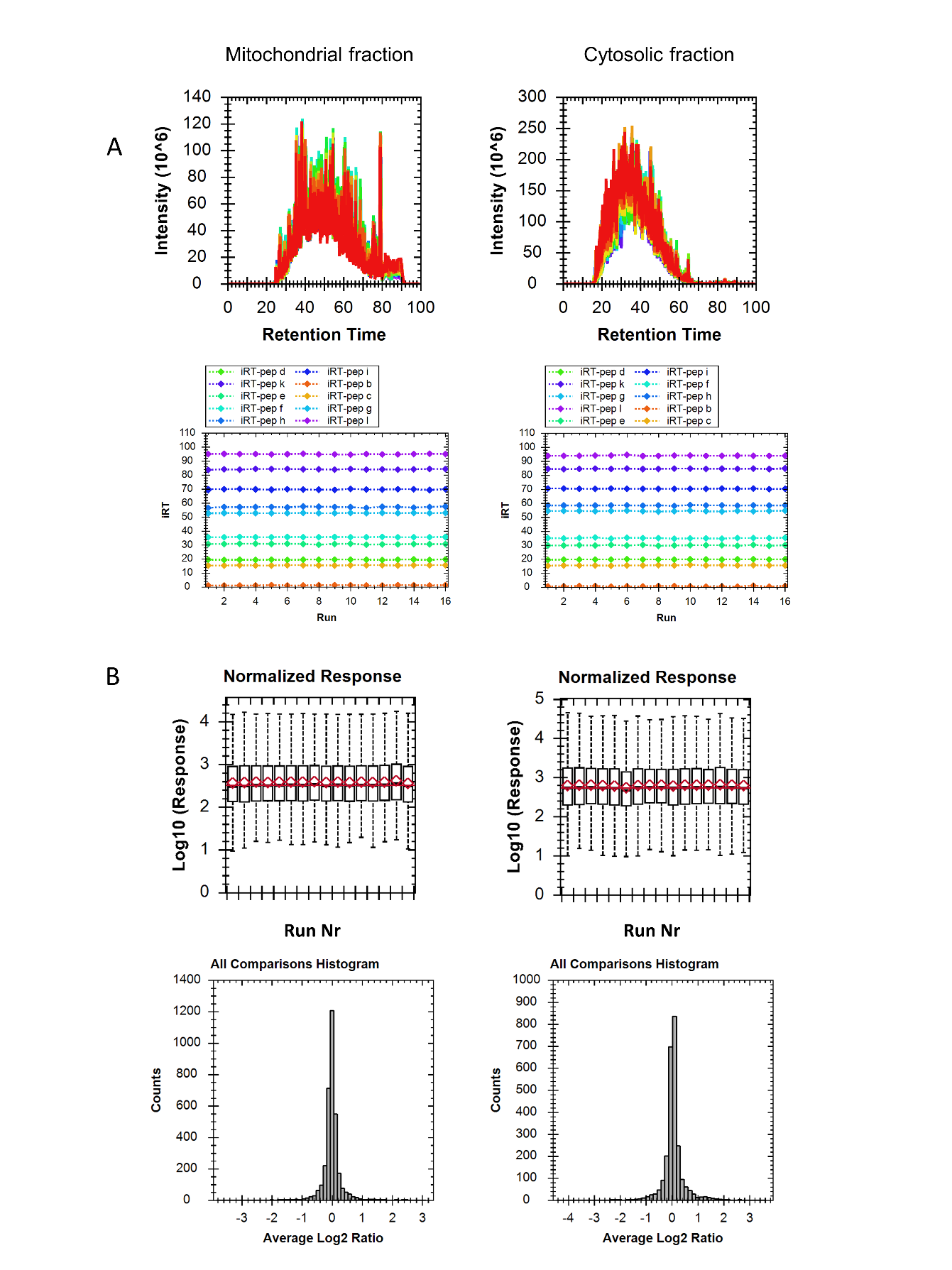

Supplement: Supplementary file 2 — Supplementary file2 (DOCX 2091 KB) [file 11356_2022_22179_MOESM2_ESM.docx]
